# Results from the Tube Versus Trabeculectomy Study

**DOI:** 10.4103/0974-9233.56219

**Published:** 2009

**Authors:** Steven J. Gedde

**Affiliations:** Bascom Palmer Eye Institute, Miller School of Medicine, University of Miami, Miami, Florida, USA

**Keywords:** Trabeculectomy, tube shunt, Tube Versus Trabeculectomy Study

## Abstract

The Tube Versus Trabeculectomy (TVT) Study is a multicenter randomized clinical trial comparing the safety and efficacy of tube shunt surgery to trabeculectomy with mitomycin (MMC) in eyes with previous cataract and/or failed glaucoma surgery. Tube shunt surgery was more likely to maintain intraocular pressure (IOP) control and avoid persistent hypotony, reoperation for glaucoma, or loss of light perception vision than trabeculectomy with MMC during the first year of follow-up. Both surgical procedures had similar IOP reduction at 1 year, but less supplemental medical therapy was used following trabeculectomy. The incidence of postoperative complications was higher after trabeculectomy with MMC relative to tube shunt surgery, but serious complications associated with vision loss and/or reoperation developed with similar frequency after both of the procedures. There was no significant difference in the rate of vision loss following trabeculectomy with MMC and tube shunt surgery after 1 year of follow-up. Cataract progression was common, but occurred with similar frequency with both of the surgical procedures.

## INTRODUCTION

Recently, practice patterns in glaucoma surgery have undergone significant changes. The number of trabeculectomies performed among Medicare beneficiaries between 1995 and 2004 has steadily declined, and there has been a concurrent rise in tube shunt surgery.^1^ Surveys of the American Glaucoma Society membership has also demonstrated an increase in the popularity of tube shunts as an alternative to trabeculectomy, especially in eyes that have undergone previous ocular surgery.^2^,^3^ Despite these trends, there is no clear consensus among glaucoma specialists regarding the preferred surgical approach for managing glaucoma in eyes that have previously undergone cataract or glaucoma surgery, with some surgeons favoring a trabeculectomy with an adjunctive antifibrotic agent and others preferring a tube shunt.^2^,^3^

The Tube Versus Trabeculectomy (TVT) Study is a multicenter randomized clinical trial comparing the safety and efficacy of tube shunt surgery to trabeculectomy with mitomycin C (MMC) in eyes that have undergone prior ocular surgery. Patients with uncontrolled glaucoma who had previously undergone cataract extraction with intraocular lens implantation and/or failed trabeculectomy were randomized to receive a 350-mm^2^ Baerveldt glaucoma implant or trabeculectomy with MMC. The goal of this investigator initiated study is to provide information that will assist in surgical decision making in similar patient groups.

## MATERIAL AND METHODS

**Figure 1:**
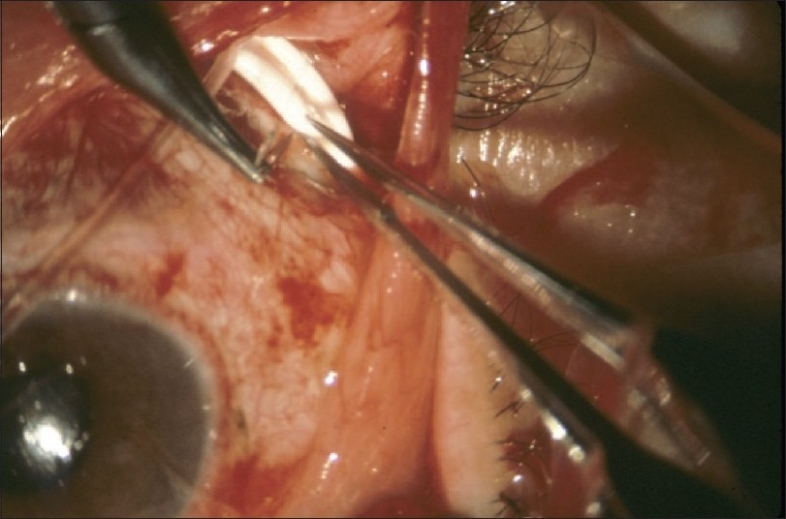
Intraoperative photograph of tube shunt surgery

**Figure 2:**
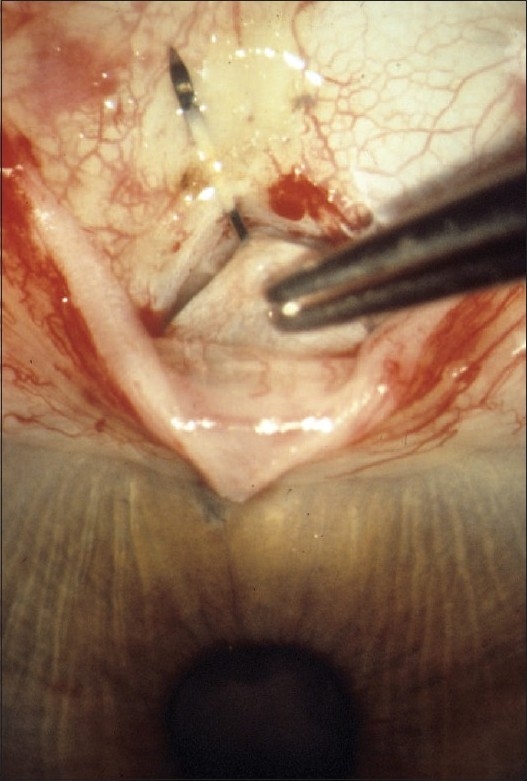
Intraoperative photograph of trabeculectomy with mitomycin C

The design and methods of the TVT Study were previously described in detail.^4^ The study is registered with http://www.clinicaltrials.gov (NCT00306852). The inclusion and exclusion criteria for the study are listed in Table 1. Enrolled patients were randomly assigned to treatment with a tube shunt or trabeculectomy with MMC. Patients in the tube group underwent placement of a 350-mm^2^ Baerveldt glaucoma implant superotemporally with a complete restriction of flow at the time of implantation [Figure 1]. Patients in the trabeculectomy group had a superior trabeculectomy with a standard dosage of MMC of 0.4 mg/ml for 4 min [Figure 2]. Follow-up visits were scheduled 1 day, 1 week, 1 month, 3 months, 6 months, 1 year, 18 months, 2 years, 3 years, 4 years, and 5 years postoperatively. Each examination included measurement of Snellen visual acuity (VA), intraocular pressure (IOP), slit lamp biomicroscopy, Seidel testing, and ophthalmoscopy. Humphrey perimetry, Early Treatment Diabetic Retinopathy Study (ETDRS) VA, and quality of life using the National Eye Institute Questionnaire (NEI VFQ-25) were assessed at baseline and at the annual follow-up visits. A formal motility evaluation was performed in all patients at baseline and at the 1-year and 5-year follow-up visits and at any visit after 3 months in which the patient reported diplopia. The examining clinician provided a reason for loss of 2 or more lines of Snellen VA at follow-up visits after 3 months.

**Table 1:** Patient eligibility criteria in the Tube Versus Trabeculectomy Study

Inclusion criteria	Age 18 to 85 years
	Inadequately controlled glaucoma with IOP ≥ 18 mm Hg and ≤ 40 mm Hg on maximum tolerated medical therapy
	Previous cataract extraction with intraocular lens implantation, trabeculectomy, or both
Exclusion criteria	Unwilling or unable to give consent, unwilling to accept randomization, or unable to return for scheduled protocol visits
	Pregnant or nursing women
	No light perception vision
	Active iris neovascularization or active proliferative retinopathy
	Iridocorneal endothelial syndrome
	Epithelial of fibrous downgrowth
	Aphakia
	Vitreous in the anterior chamber for which a vitrectomy is anticipated
	Chronic or recurrent uveitis
	Severe posterior blepharitis
	Unwilling to discontinue contact lens use after surgery
	Previous cyclodestructive procedure, scleral buckling procedure, or silicone oil present
	Conjunctival scarring precluding a trabeculectomy superiorly
	Need for glaucoma surgery combined with other ocular procedures (i.e. cataract surgery, penetrating keratoplasty, or retinal surgery) or anticipated need for additional ocular surgery

Failure was prospectively defined as IOP > 21 mm Hg or not reduced by 20% below baseline on two consecutive follow-up visits after 3 months, IOP ≤ 5 mm Hg on two consecutive follow-up visits after 3 months, reoperation for glaucoma, or loss of light perception vision. Reoperation for glaucoma or a complication was defined as additional surgery requiring a return to the operating room. Cyclodestruction was also counted as a reoperation for glaucoma, and a vitreous tap with the injection of intravitreal antibiotics was a reoperation for a complication. Interventions performed at the slit lamp, such as needling procedures or reformation of the anterior chamber, were not considered reoperations. Serious complications were defined as surgical complications that were associated with loss of two or more lines of Snellen VA and/or reoperation to manage the complication.

## RESULTS

A total of 212 eyes of 212 patients were enrolled at 17 Clinical Centers, including 107 in the tube group and 105 in the trabeculectomy group.^4^ The mean age of the study population was 71.0 years, and 53% were women. The baseline IOP was 25.3 ± 5.3 mm Hg (mean ± SD) on 3.1 ± 1.2 glaucoma medications (mean ± SD), and 81% of patients had primary open-angle glaucoma. There were no significant differences in any of the demographic and ocular characteristics between the tube group and the trabeculectomy group, suggesting that randomization was very effective in creating two balanced treatment groups.

Postoperative interventions in the tube group included rip cord removal in 19 (18%) patients, laser suture lysis in 5 (5%) patients, anterior chamber reformation in 4 (4%) patients, needling procedures in 2 (2%) patients, and injection of 5-fluorouracil in 1 (1%) patient.^5^ In the trabeculectomy group, laser suture lysis was performed in 51 (49%) patients, 5-fluorouracil injection in 23 (22%) patients, needling procedures in 8 (8%) patients, injection of intracameral tissue plasminogen activator in 2 (2%) patients, and suturing of a wound leak in 1 (1%) patient.^5^

**Figure 3:**
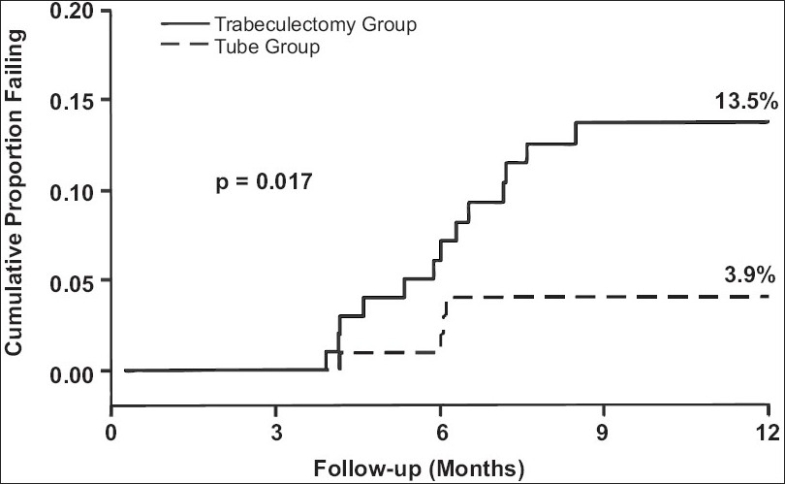
Kaplan-Meier plots of the probability of failure in the Tube Versus Trabeculectomy (TVT) Study

Table 2 presents the IOPs and number of glaucoma medications at baseline and 1 year. The trabeculectomy group had significantly lower mean IOPs than the tube group at all follow-up visits during the first 3 months, but there was no significant difference in the degree of IOP reduction between the treatment groups after 3 months.^6^ There was a significantly increased use of adjunctive medical therapy in the tube group in comparison to the trabeculectomy group at all follow-up visits during the first postoperative year.^6^ Kaplan-Meier plots of the probability of failure are shown in Figure 3. The cumulative probability of failure was 3.9% in the tube group and 13.5% in the trabeculectomy group at 1 year, a difference that was statistically significant (p = 0.017, log rank test).^6^ A greater proportion of patients in the trabeculectomy group (5%) required reoperations for glaucoma in comparison to those of the tube group (1%) during the first year of follow-up. However, this difference did not quite reach the level of statistical significance (p = 0.086, log rank test).^6^

**Table 2:** Intraocular pressure and medical therapy in the Tube Versus Trabeculectomy (TVT) Study

	Tube Group	Trabeculectomy Group	*P* value
Baseline			
IOP (mm Hg)	25.1 ± 5.3	25.6 ± 5.3	0.56
Glaucoma medications	3.2 ± 1.1	3.0 ± 1.2	0.17
1 year			
IOP (mm Hg)	12.4 ± 3.9	12.7 ± 5.8	0.73
Glaucoma medications	1.3 ± 1.3	0.5 ± 0.9	< 0.001

Data are presented as mean ± standard deviation. IOP = intraocular pressure

There was reduction in VA in both the tube group and the trabeculectomy group during the first year of follow-up, but Snellen and ETDRS VA were similar between treatment groups at 1 year.^6^ There was no difference in the rate of loss of two or more lines of Snellen VA between the tube group (32%) and the trabeculectomy group (33%) after 1 year (p = 1.00, chi-square test).^6^ Many causes of vision loss were not directly attributable to the surgical procedures under study, such as macular degeneration and posterior capsular opacification.

There were a large number of surgical complications in the TVT Study, but most were transient and self-limited. A similar rate of intraoperative complications was observed in the tube group (7%) and the trabeculectomy group (10%) (p = 0.59, chi-square test).^5^ Significantly more patients in the trabeculectomy group (57%) experienced postoperative complications than those in the tube group (34%) during the first year of follow-up (p = 0.001, chi-square test).^5^ However, all complications are not equal in severity, and serious complications associated with reoperation to manage the complication and/or loss of two Snellen lines of vision occurred with similar frequency in the trabeculectomy group (27%) and tube group (17%) at 1 year (p = 0.12, chi-square test).^5^ Wound leaks (p = 0.004, chi-square test) and dysesthesia (p = 0.034, Fischer's exact test) were significantly more common in the trabeculectomy group than the tube group.^5^ New postoperative motility disturbances developed more frequently in the tube group (9.9%) than the trabeculectomy group (0%) during the first year of follow-up (p = 0.005, Fisher's exact test), and there was a tendency for diplopia to occur more commonly in the tube group (5%) than the trabeculectomy group (0%) (p = 0.06, Fisher's exact test).^7^

Cataract progression was common among phakic patients in the TVT Study, but occurred with similar frequency in the tube group (33%) and the trabeculectomy group (48%) during the first year of follow-up (p = 0.50, chi-square test).^5^ There was no statistical difference in the rate of cataract surgery between the tube group (17%) and the trabeculectomy group (5%) after 1 year (p = 0.35, Chi-square test).^5^

## DISCUSSION

The TVT Study enrolled patients with medically uncontrolled glaucoma who had previous cataract extraction with intraocular lens implantation and/or failed filtering surgery and randomized them to surgical treatment with a trabeculectomy with MMC or tube shunt placement. During the first year of follow-up, patients who were treated with tube shunt surgery were more likely to maintain IOP control and avoid persistent hypotony, loss of light perception vision, or reoperation for glaucoma in comparison to those who underwent trabeculectomy with MMC. Both surgical procedures were associated with similar IOP reduction at 1 year, but there was less need for supplemental medical therapy after trabeculectomy. The incidence of postoperative complications was higher after trabeculectomy with MMC as compared with tube shunt surgery. However, serious complications associated with reoperation and/or vision loss occurred with similar frequency after both of the surgical procedures. After 1 year of follow-up, there was no significant difference in the rate of vision loss following trabeculectomy with MMC or tube shunt surgery. Cataract progression was common, but occurred with similar frequency with both surgical procedures.

The results of the TVT Study provide further evidence that the role of tube shunts in the surgical management of glaucoma should be expanded. Although these devices have historically been reserved for refractory glaucomas at high risk of failure with standard filtering surgery, this study enrolled eyes at lower risk for failure. The TVT Study does not demonstrate clear superiority of one glaucoma operation over the other. There are other factors that must be considered when selecting a surgical procedure, including the surgeon's skill and experience with both operations, the patient's willingness to undergo repeat glaucoma surgery, and the planned surgical approach if failure occurs. Because postoperative interventions and surgical complications were more common after trabeculectomy, tube shunt surgery may be the preferred surgical approach when follow-up is limited. Additional data will be forthcoming from the TVT Study, and it is needed to fully assess the risks and benefits of tube shunt surgery and trabeculectomy with MMC in managing medically uncontrolled glaucoma in similar patient groups.

## APPENDIX

**Participating Centers and Committees in the Tube Versus Trabeculectomy Study**

**Clinical Centers:**

**Bascom Palmer Eye Institute, Miller School of Medicine, University of Miami (Miami, Florida USA):** Principal Investigator: Steven Gedde, M.D.; Coinvestigators: Douglas Anderson, M.D., Donald Budenz, M.D., M.P.H., Madeline Del Calvo, Francisco Fantes, M.D., David Greenfield, M.D., Elizabeth Hodapp, M.D., Richard Lee, M.D., Ph.D., Alexia Marcellino, Paul Palmberg, M.D. Ph.D., Richard Parrish II, M.D.

**Duke University (Durham, North Carolina USA):** Principal Investigator: Leon Herndon, M.D.; Coinvestigators: Pratap Challa, M.D., Cecile Santiago-Turla, M.D.

**Indiana University (Indianapolis, Indiana USA):** Principal Investigator: Darrell WuDunn, M.D., Ph.D.

**Loyola University (Maywood, Illinois USA):** Principal Investigator: Geoffrey Emerick, M.D.

**Medical College of Wisconsin (Milwaukee, Wisconsin USA):** Principal Investigator: Dale Heuer, M.D.

**Medical University of South Carolina (Charleston, South Carolina USA):** Principal Investigator: Alexander Kent, M.D.; Coinvestigators: Carol Bradham, Lisa Langdale.

**Moorfields Eye Hospital (London, England):** Principal Investigator: Keith Barton, M.D.; Coinvestigator: Francesca Amalfitano, Poornima Rai, M.D.

**New York Eye and Ear Infirmary (New York, New York USA):** Principal Investigator: Paul Sidoti, M.D.; Coinvestigators: Amy Gedal, James Luayon, Roma Ovase, Katy Tai

**Scripps Clinic (La Jolla, California USA):** Principal Investigator: Quang Nguyen, M.D.; Coinvestigator: Neva Miller

**St. Louis University (St. Louis Missouri USA):** Principal Investigator: Steven Shields, M.D.; Coinvestigators: Kevin Anderson, Frank Moya, M.D.

**University of California Davis (Sacramento, California USA):** Principal Investigator: James Brandt, M.D.; Coinvestigators: Michele Lim, M.D., Marilyn Sponzo.

**University of Florida (Gainesville, Florida USA):** Principal Investigator: Mark Sherwood, M.D.; Coinvestigator: Revonda Burke

**University of Oklahoma (Oklahoma City, Oklahoma USA):** Principal Investigator: Gregory Skuta, M.D.; Coinvestigators: Jason Jobson, Lisa Ogilbee, Adam Reynolds, M.D., Steven Sarkisian, Jr., M.D.

**University of Southern California (Los Angeles, California USA):** Principal Investigator: Rohit Varma, M.D., M.P.H.; Coinvestigators: Brian Francis, M.D., Frances Walonker

**University of Texas Houston (Houston, Texas USA):** Principal Investigator: Robert Feldman, M.D.; Coinvestigators: Laura Baker, Nicholas Bell, JoLene Carranza, Athena Espinoza

**University of Virginia (Charlottesville, Virginia USA):** Principal Investigator: Bruce Prum, M.D.; Coinvestigator: Janis Beall

**University of Wisconsin (Madison, Wisconsin USA):** Principal Investigator: Todd Perkins, M.D.; Coinvestigators: Paul Kaufman, M.D., Tracy Perkins, Barbara Soderling.

**Statistical Coordinating Center, Bascom Palmer Eye Institute, Miller School of Medicine, University of Miami (Miami, Florida USA):** William Feuer, M.S., Luz Londono, Joyce Schiffman, M.S., Wei Shi, M.S.

**Safety and Data Monitoring Committee:** Philip Chen, M.D., William Feuer, M.S., Joyce Schiffman, M.S., Kuldev Singh, M.D., M.P.H., George Spaeth, M.D., Martha Wright, M.D.

**Steering Committee:** Keith Barton, M.D., James Brandt, M.D., Geoffrey Emerick, M.D., Robert Feldman, M.D., Steven Gedde, M.D., Leon Herndon, M.D., Dale Heuer, M.D., Alexander Kent, M.D., Quang Nguyen, M.D., Richard Parrish II, M.D., Todd Perkins, M.D., Bruce Prum, M.D., Mark Sherwood, M.D., Steven Shields, M.D., Paul Sidoti, M.D., Gregory Skuta, M.D., Rohit Varma, M.D., M.P.H., Darrell WuDunn, M.D., Ph.D.

**Study Chairmen:** Steven Gedde, M.D., Dale Heuer, M.D., Richard Parrish II, M.D.
